# Sampling methods for renewable gases and related gases: challenges and current limitations

**DOI:** 10.1007/s00216-022-03949-0

**Published:** 2022-02-07

**Authors:** Karine Arrhenius, Lorena Francini, Oliver Büker

**Affiliations:** Research Institutes of Sweden AB (RISE), Frans Perssons väg, 412 76 Gothenburg, Sweden

**Keywords:** Renewable gases, Sampling, Material compatibility

## Abstract

Renewable gases, hydrogen and biomethane can be used for the same applications as natural gas: to heat homes, power vehicles and generate electricity. They have the potential to contribute to the decarbonisation of the gas grid. Hydrogen blending with existing natural gas pipelines is also proposed as a means to increase the performance of renewable energy systems. Carbon capture and storage (CCS) and carbon capture and utilisation (CCU) technologies can be an answer to the global challenge of significantly reducing greenhouse gas emissions. Due to production methods, these gases typically contain species in trace amounts that can negatively impact the equipment they come into contact with or pipelines when injected into the gas grid. It is therefore necessary to ensure proper (and stable) gas quality that meets the requirements set out in the relevant standards. The gas quality standards require the collection and transport of a representative gas sample from the point of use to the analytical laboratory; i.e., no compounds may be added to or removed from the gas during sampling and transport. To obtain a representative sample, many challenges must be overcome. The biggest challenge is material compatibility and managing adsorption risks in the sampling systems (sampling line and sampling vessels). However, other challenges arise from the need for flow measurement with non-pure gases or from the nature of the matrix. Currently, there are no conclusive results of short-term stability measurements carried out under gas purity conditions (suitable pressure, matrix, appropriate concentrations, simultaneous presence of several species).

## Introduction

Renewable gas is the term used to describe gases that can be used as a clean energy source, i.e. whose combustion does not produce any additional emissions. There are two main forms: renewable hydrogen and biomethane. Hydrogen [1] and biomethane [2] have the same applications as natural gas: to heat homes, power vehicles and generate electricity. Both have the potential to contribute to the decarbonisation of the gas grid.

There are several ways to produce renewable hydrogen: through electrolysis using electricity from renewable sources (e.g. wind), by biomass conversion—either thermochemical or biochemical conversion into intermediates that can then be separated or reformed into hydrogen, or by dark fermentation techniques that directly produce hydrogen, or by solar conversion—either thermolysis, using solar heat for high-temperature hydrogen production in the chemical cycle, or photolysis, using solar photons in biological or electrochemical systems to directly produce hydrogen [3].

Biomethane is a nearly pure source of methane produced either by “upgrading” biogas (a process in which most of the carbon dioxide is removed from the biogas along with other impurities) or by gasifying solid biomass followed by methanation [4].

Biogas is produced by decomposing organic matter under anaerobic conditions so that biogas with a methane content of 50–60% can be obtained. The biogas is then purified, and this process is usually referred to as upgrading the biogas to biomethane (with a methane content of over 95%). During the upgrading process, a carbon dioxide–containing stream is also produced.

Different fuels emit different amounts of carbon dioxide (CO_2_) in relation to the energy they produce during combustion [5] (however, the combustion of hydrogen only produces water vapour and energy in the form of heat, with no carbon emissions). Capturing CO_2_ can lead to negative CO_2_ emission. Carbon capture and storage (CCS) refers to technologies that focus on selectively removing CO_2_ from gas streams [6] (usually from large point sources such as a cement factory or biomass power plant [7]), compressing it to a supercritical state and finally transporting and sequestering it in geological formations, including depleted oil and gas reservoirs or oceans [8].

Another alternative is carbon capture and utilisation (CCU) technologies, where captured CO_2_ is converted into valuable products as a renewable carbon feedstock instead of being permanently sequestered. CO_2_ is often used as a feedstock for various applications, such as carbonated beverage production, food preservation, urea production, water treatment, enhanced oil recovery, chemical production and polymer production [9, 10]. As microorganisms (such as algae) are naturally capable of capturing CO_2_ and converting it into chemicals and fuels [11], the biological use of CO_2_ provides another route to the production of biodiesel and various biomass-derived chemical feedstocks (used as food, silage, biogas and fertiliser) [12].

Due to the production methods (in the case of hydrogen and biomethane) or the origin (in the case of carbon dioxide), these gases usually contain species in traces that can have a negative impact on the equipment they come into contact with or on the pipelines when they are injected into the gas network. For this reason, and depending on the area of application of hydrogen, biomethane and carbon dioxide, a certain gas quality is generally required. This has led to the development of several standards that contain requirements for fuel quality assessment for different applications. Examples of such standards are EN17124 [13], ISO14687 [14] for hydrogen used as fuel in fuel cell vehicles EN16723 standards [15, 16] for biomethane used in transport and injected into the natural gas grid, and ISO TR 27921 [17] for carbon dioxide capture, transport and geological storage.

Each of these standards requires analysis in a laboratory and therefore requires the collection and transport of a gas sample from the point of use. The sample taken must be representative of the gas supplied; this assumes that no compounds are added to or removed from the gas during sampling. However, it is known that adsorption effects in the sampling vessel or sampling line can lead to a loss of the impurities to be analysed. The purpose of this study is to discuss the challenges and current limitations of the sampling methods used for these three gases.

## Sampling vessels

Different vessels can be used for sampling renewable gas (hydrogen and biomethane) and other related gases (e.g. carbon dioxide).

There are two basic ways of sampling, with or without enrichment [18]. For sampling with enrichment, the desired compounds are adsorbed onto a solid collection phase (with adsorption tubes) or absorbed into a solution (with impingers) while the matrix passes without retention. For sampling without enrichment, bags, canisters or cylinders made of different materials are used with or without treatment or passivation technologies. With these vessels, no distinction is made between the individual compounds during collection; thus, all compounds are collected. The choice of vessels depends on many parameters, including the pressure and temperature of the gas at the sampling point, safety aspects, requirements/recommendations in standards, transport regulations and data from storage stability studies. For sampling hydrogen for quality assessment according to ISO14687 and ISO21087 [19], samples should be taken at the hydrogen filling station (HRS) nozzle, where the pressure is either 350 or 700 bar.

Sampling in cylinders is almost a requirement for safety reasons. ISO21087 also states that transfer or sampling from the original vessel should be avoided (but is not prohibited) to minimise the risk of impurity losses and contamination. Sampling in bags or on sorption tubes is more commonly used for biogas and biomethane, as most biogas and biomethane production plants operate at low pressure (< 4 bar). Guidelines for sampling biomethane in bags and on sorbent hoses will be added in the revision of the of the ISO10715 standard [20].

## Challenges

The main challenge when sampling gases for species at trace levels is managing the risk of loss of contaminants by adsorption on the walls of bags, cylinders or canisters and on the media (partial adsorption or irreversible adsorption for pipes) or by reaction (chemical reaction between species or between species and the matrix). The risk of adsorption in the sampling line is another challenge. Other challenges arise from the need for flow measurement specifically for the enrichment methods and especially for biomethane, hydrogen-enriched natural gas and even carbon dioxide, the exact composition of which may not be known until it has been fully analysed in a laboratory.

### Biomethane

The EN 16,723 standard sets requirements for a number of parameters, including the content of trace substances such as siloxanes and sulphur compounds. However, depending on the substrate being fermented and the treatment method, biomethane may also contain other volatile organic compounds (VOCs) such as terpenes [21], hydrocarbons, oxygenated hydrocarbons, halogenated hydrocarbons and nitrogenous compounds, and it may be relevant to analyse these species as well. One of the obvious challenges is the wide range of boiling points, e.g. in the case of halogenated hydrocarbons, where species found in biogas samples have boiling points ranging from − 82 °C (CHF3) to over 300 °C (e.g. C6Cl6, 325 °C), but also the fact that species also differ in terms of polarity, water solubility and reactivity.

**Fig. 1:**
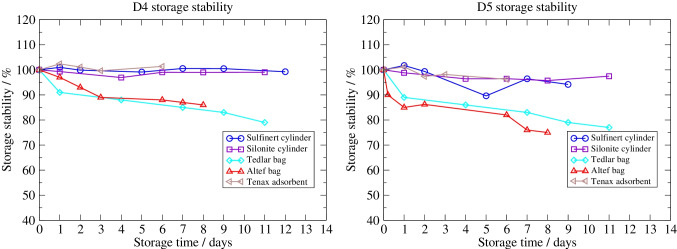
Short-term storage stability in cylinders, bags and on adsorbent for siloxanes D4 and D5 (figure from Arrhenius et al. [22])

**Fig. 2:**
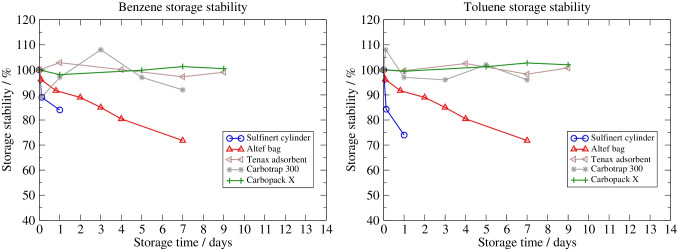
Short-term storage stability in cylinders, bags and on adsorbent for benzene and toluene (figure from Arrhenius et al. [22])

Short-term stability studies [22] for siloxanes, halogenated compounds and BTEX (benzene, toluene, ethylbenzene and xylenes) in cylinders, bags and onto sorbent tubes were carried out as part of the EMRP project ENG54 “Metrology for biogas” [23] as shown in Figs. 1 and 2. The study showed that at least, concentration, pressure and the presence of water affect the suitability of a vessel. While storage in gas cylinders seemed to be a reliable alternative when the gas was taken at a relatively high pressure (> 50 bar to 60 bar as in the case of siloxanes D4 and D5 in Fig. 1), adsorption effects occurred directly on the inner surface of the cylinder at low pressure (less than 10 bar as in the case of toluene in Fig. 2). The occurrence of adsorption on the walls of bags has been shown to be strongly related to the boiling point of the target species, which is consistent with information provided by bag manufacturers [24]. Compounds with a boiling point above about 150 °C tend to be more likely to be partially lost by adsorption in pouches than compounds with lower boiling points. The loss of concentration appears to be concentration dependent and to occur to a greater extent during the initial storage period when the internal surfaces become saturated [18].

Material compatibility was investigated for different cylinders as part of the EMPIR project “Metrology for biomethane” [25], with tests to determine the long-term stability (1.5 years) of siloxane standards in six different types of cylinders: Spectraseal from BOC, Spectraseal with H_2_S-treatment performed by NPL (called Spectraseal PT), Performax from Effectech, Megalife from Air Liquide, Experis from Air Liquide and PB passivation from New Energy Technology (deliverable D1). The targeted siloxanes were hexamethyldisiloxane (L2), octamethyltrisiloxane (L3), octamethylcyclotetrasiloxane (D4) and decamethylcyclopentasiloxane (D5) in a methane matrix. These tests were carried out at a pressure of 100 bar and concentrations of 55 nmol/mol for L2, 35 nmol/mol for L3, 27 nmol/mol for D4 and 22 nmol/mol for D5. In these tests, measurements were taken on different cylinders 30 days after the mixture was prepared and the results were compared with measurements taken on the day of preparation. With this time frame, these results can be used to assess material compatibility for sampling. The results for the Experis, PB and Megalife cylinders showed that all siloxanes were stable (within 5% change) compared to day 0. The results for Performax and Spectraseal PT showed that only D5 and D4 were stable within the limit of 5% change. L2 levels increased (+ 9% and + 11% change compared to day 0 for Performax and Spectraseal PT, respectively) and L3 levels decreased (− 8% and − 12% change compared to day 0 for Performax and Spectraseal PT, respectively). The results for Spectraseal showed that only D4 was stable within a 5% change. The concentrations of L2 and D5 increased (+ 9% change compared to day 0) and the concentration of L3 decreased (− 12% change compared to day 0).

Sorbent tubes contain various types of solid adsorbents. Commonly used adsorbent materials include activated carbon, silica gel and organic porous polymers such as Tenax and XAD resins [26]. Factors to consider when selecting suitable sorbents [27] include the strength of the interaction between sorbent and sorbate, which is highly dependent on the boiling point of the sorbate, temperature, artefacts, hydrophobicity and inertness (some sorbents contain chemically active substances and are generally unsuitable for reactive species—sulphur compounds, terpenes, amines, etc.). Weak sorbent [28], such as a porous polymer sorbent, should be chosen for species with a boiling point above 100 °C, a medium strong sorbent, such as a graphitised carbon black, should be chosen for species with a boiling point between 30 and 100 °C, and a strong sorbent, such as a carbon molecular sieve, should be chosen for species with a boiling point in the range − 48 to 30 °C. Finally, compounds with boiling points below − 48 °C are usually too volatile.

As shown in Figs. 1 and 2, short-term storage stability can be a problem when sampling in gas bags. When sampling onto adsorbents, the flow rate must be stable during the whole duration of the sampling, which is not always possible on site. Another challenge with direct sampling on a sorbent tube is flow measurement. Sampling on sorbent tubes usually requires collecting a known volume of gas at an appropriate flow rate on the sorbent tube, preferably with a flowmeter. Many flowmeters are calibrated for a fixed composition, and changes in composition can drastically affect the flow measurement [29].

The composition of biogas and biomethane varies in terms of the main components depending on the process. The methane content varies between 40 and 99 vol-% and the carbon dioxide content between less than 1 vol-% to 60 vol-%. Other compounds such as nitrogen, water vapour and hydrogen sulphide may be present in the gas in amounts ranging from a few µmol/mol to up to mol/mol. Finally, hydrocarbons such as ethane and propane may also be present in the gas if, for example, natural gas has been mixed with biomethane. In general, the exact composition is only known after a complete analysis in a laboratory.

**Fig. 3:**
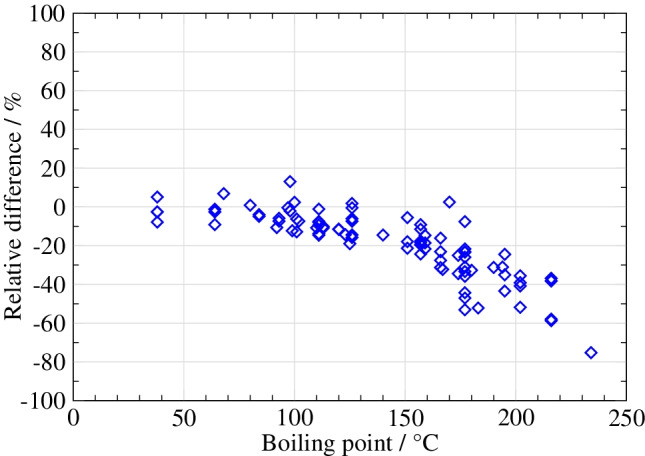
Difference in VOC concentration measured on Tenax TA after transfer from a bag using two different flows (100 ml/min versus 300 ml/min—reference) (figure from Arrhenius et al. [30])

Combining the two methods by transferring a volume of gas from the bag into a sorbent tube immediately after filling the bag has been proposed as another sampling strategy, as it could provide a solution to overcome the disadvantages associated with both methods. However, this study [30] has shown that the transfer must be done at relatively high flow rates (Fig. 3), especially if the gas contains a higher water content (as in biogas). If lower flow rates are used, the concentrations can be underestimated, which has a particular effect on compounds with higher boiling points (above 150 °C), probably due to adsorption on the walls of the bags.

The risks of adsorption at the sampling line must also be considered. Tests [31] carried out as part of the EMRP ENG54 “Metrology for biogas” project have shown that adsorption effects occur even within the regulator of a cylinder filled with 90 bar of a reference material consisting of four siloxanes (L2 with a boiling point of 100 °C, L3 with a boiling point of 153 °C, D4 with a boiling point of 176 °C and D5 with a boiling point of 210 °C) in a mixture of methane, carbon dioxide and nitrogen.

**Fig. 4:**
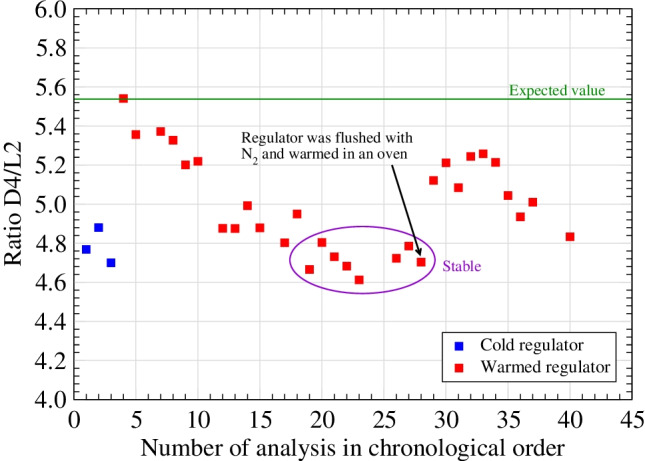
Adsorption effects in the regulator observed for D4

The samples from the cylinders were collected on sorbent tubes once the pressure had been reduced from 100 bar to just over 1 bar. In the first tests, it was found that the concentrations of D4 and D5 were much lower than expected, while the concentrations of L2 and L3 were as expected. It was suspected that adsorption effects in the regulator for D4 and D5 were the cause. To test this hypothesis, a number of samples were taken while the regulator was heated, and it was found that the concentrations of D4 and D5 were much closer to the expected concentration. However, the positive effect of heating the regulator was found to decrease with time, as shown in Fig. 4.

### Hydrogen

The EN17124 and ISO14687 standards set quality requirements for hydrogen as a fuel, including limits for a large number of gaseous species, some of which are very demanding. Another challenge is the need for “total” measurements such as total hydrocarbons, total sulphur compounds and halogenated compounds at stringent threshold limits. In addition, some of these substances are reactive or “sticky” (formaldehyde, formic acid, sulphur compounds, water, ammonia). Due to safety aspects, the sampling strategies for taking a hydrogen sample at the nozzle of a hydrogen filling station (HRS) use cylinders made of aluminium or stainless steel. These materials are more or less susceptible to absorbing reactive compounds on their surfaces. Most of the reactions that take place on the inner surface of an aluminium cylinder are cavity reactions catalysed by the aluminium oxide structure. Reactive compounds are absorbed on stainless steel by chemisorption [32].

The stability of these reactive species in different cylinders must be evaluated before selecting the appropriate cylinder. However, there is currently a lack of meaningful results from short-term stability studies conducted under conditions typical of hydrogen purity (appropriate pressure, matrix, appropriate concentrations, simultaneous presence of multiple species). So far, to our knowledge, there are no studies that have addressed these issues for species such as formaldehyde, formic acid and water (at low µmol/mol values). For sulphur compounds, especially hydrogen sulphide, there is some information available.

Most studies on hydrogen sulphide show that some treatment of stainless steel or aluminium cylinders is required. For example, one study showed that hydrogen sulphide at a concentration of 17 nmol/mol [33] was completely lost after 1 day when stored in untreated stainless steel cylinders, while the hydrogen sulphide concentration remained stable for at least 7 days when stored in SilcoNert 2000 coated cylinders. Another study has shown that 1.5 nmol/mol hydrogen sulphide in air remained stable in SilcoNert-2000-treated canisters [34].

No stability test results could be found for hydrogen sulphide at low nmol/mol levels in aluminium cylinders, but the stability of hydrogen sulphide at 500 nmol/mol in synthetic air was studied in different types of aluminium cylinders [35] (superior gas stability—SGS cylinders, basic -B aluminium alloy cylinders and acid washed—AW cylinders), with several cylinders tested for each type. Again, losses were observed for AW cylinders (total loss after 2 days) and for B cylinders (total loss after 10 days for most cylinders tested), while the hydrogen sulphide concentration remained more stable for SGS cylinders. However, some loss (up to 20% for one cylinder) was also observed for these cylinders, especially at the beginning of the tests.

In a study conducted as part of the EMPIR 16ENG01 “Metrology for hydrogen vehicles” project [36], the stability of hydrogen sulphide in a hydrogen matrix at 40 nmol/mol was compared in different types of cylinders: untreated 10-l aluminium cylinders, Spectraseal-treated 10-l aluminium cylinders, 1-l untreated stainless steel aluminium cylinders, 1-l Sulfinert treated stainless steel cylinders and 1-l Dursan stainless steel cylinders. The results in the aluminium cylinders showed a significant initial loss in both types. After this initial loss, the level of hydrogen sulphide (H_2_S) in both types of sampling vessels remained stable over the test period (5 weeks, Fig. 4).

**Fig. 5:**
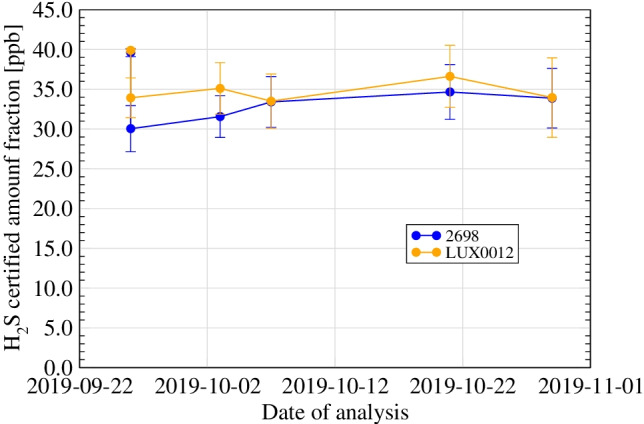
Short-term stability study for H_2_S at 40 nmol/mol in H_2_ (Spectraseal-treated aluminium sampling vessel (2698) and untreated aluminium sampling vessel (LUX0012)) (figure from Arrhenius et al. [36])

**Fig. 6:**
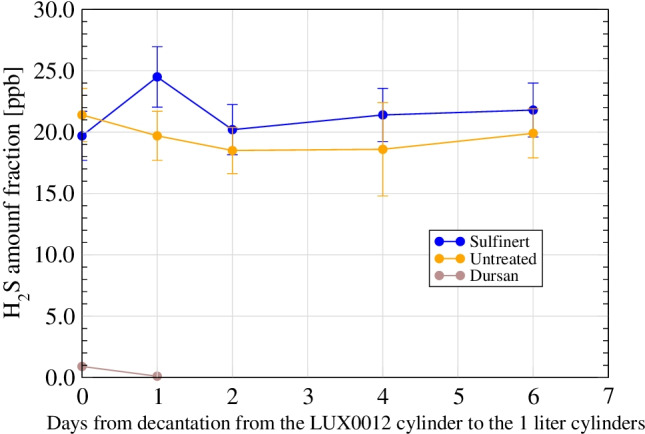
Short-term stability study for H_2_S at around 40 nmol/mol in H_2_ after decantation to 1-l stainless steel cylinders)—test 3 (figure from Arrhenius et al. [36])

The stainless steel cylinders were filled 6 months after the preparation of the mixture by decanting from the untreated aluminium cylinders. The results (Figs. 5 and 6) show an almost complete loss when decanting into the Dursan cylinder, while H_2_S remained stable for a period of at least 6 days after decanting in both the untreated and the Sulfinert cylinders. The concentration in these cylinders was significantly lower than the concentration measured in the original aluminium cylinder (40 nmol/mol). This may be due to the time laps between the preparation of the original mixture and decanting (6 months), but also to initial losses in the stainless steel cylinders.

Some initial losses were observed for tests performed at 10 times higher than the threshold value of ISO14687 [37] (4 nmol/mol). It is likely that larger losses could occur at even lower amount fractions (closer to the threshold).

Many compounds belonging to the total species are organic. Therefore, the use of sorption tubes could be an alternative sampling method for the total species, as it not only provides a sum of the concentrations, but also an identification of the compound(s) actually present in the hydrogen. However, due to the wide range of boiling points of compounds in these families, the existing sorbent tubes are not universal enough to capture all possible impurities. In a recent study [38], the short-term stability (over one week) was determined for a number of compounds belonging to the three total families on different sorbent materials. The most suitable sorbent appeared to be a three-bed sorbent containing a weak (Tenax TA), a medium (Carbograph 1TD) and a strong sorbent (Carboxen 1003) that can be used for the three families.

It has also been shown that it may be necessary to use a combination of small sampling volumes (to reduce the risk of volatiles breaking through the sorbent bed during sampling) and larger sampling volumes (to achieve the detection limit required by ISO14687, especially for sulphur compounds). In addition, this study has shown that compounds retained on the sorbents can be analysed several days after sampling without any significant change in their concentration, except for species such as vinyl chloride, chloroform and ethanol.

As the pressure has to be reduced to a maximum of 10 bar (and preferably lower), sampling on the tubes at the nozzle would be a major challenge in many respects, including safety aspects. The possibilities of using vented hydrogen need to be evaluated. Transferring the hydrogen sample from the sampling cylinder to the sorbent tubes is not prohibited, although there is an increased risk of losing impurities and further contaminating the sample. However, this is currently the best option for sampling hydrogen on sorption tubes. Some of these aspects are being further investigated in the EMPIR 19ENG04 “Metrology for hydrogen vehicles 2” project [39].

The results of the available stability studies as well as experience from industry are summarised in a table in a recent article [32], which is reproduced here (Table 1).

**Table 1:** Cylinder suitability for a time period of 4 months (table from Arrhenius et al. [32])

	Stainless steel				Aluminium									
	Untreated		Sulfinert®		Untreated		Aculife VII		Performax		SPECTRA-SEAL		Untreated SGS	
	a	b	a	b	a	b	a	b	a	b	a	b	a	b
C_2_H_6_	X	X	X	X	X	X	X	X	X	X	S	S	S	S
He	X	X	X	X	X	X	X	X	X	X	S	S	S	S
N_2_	X	X	X	X	X	X	X	X	X	X	S	S	S	S
Ar	X	X	X	X	X	X	X	X	X	X	S	S	S	S
CO_2_	X	X	X	X	X	X	X	X	X	X	S	S	S	S
CO	i.d	S	i.d	S	S	S	i.d	i.d	i.d	i.d	S	S	S	S
H_2_S	i.d	I/S	X	S	i.d	I	i.d	I	i.d	i.d	I	I	S	i.d
HCl	i.d	i.d	i.d	I	i.d	i.d	i.d	I	i.d	i.d	i.d	i.d	i.d	i.d
CH_2_O	i.d	i.d	i.d	S*	i.d	i.d	i.d	i.d	i.d	S*	I	I	I	i.d
CH_2_OH	i.d	i.d	i.d	i.d	i.d	X	i.d	i.d	i.d	i.d	S	S	I	i.d
NH_3_	i.d	i.d	i.d	X	i.d	i.d	i.d	i.d	i.d	i.d	I	X	I	i.d
O_2_	i.d	i.d	i.d	i.d	i.d	i.d	i.d	i.d	i.d	i.d	S^1^	S^1^	S^1^	S^1^
H_2_O	i.d	i.d	X^2^	X^2^	i.d	i.d	i.d	i.d	i.d	i.d	S^2^	S^2^	S^2^	S^2^

a, at ISO14687:2019 threshold; b, at higher concentrations (i.e. 50 times ISO14687); X, should be suitable; S, suitability demonstrated (* more than 80% stability); I, issues were found (ex. of issues: need careful selection of the cylinder, initial loss…)

*i.d.* insufficient data

^1^Oxygen stability seems to vary between cylinders of the same internal treatment

^2^Oxygen reactivity may affect the amount fraction of water through the reaction in hydrogen matrix

As can be clearly seen from the table, in a number of cases, the data are insufficient to assess the compatibility of the materials and further studies are therefore needed to complete the table.

### Carbon dioxide

ISO Technical Committee TC265 is currently developing standards and technical reports on CO_2_ capture, transport and geological storage, including technical report ISO/TR27921:2020 [40]. Depending on the feedstock and process, CO_2_ streams captured from different sources contain various species at trace levels such as benzene, methanol, methane, carbon monoxide, hydrogen, water, ammonia, oxygen, sulphur compounds (such as H_2_S and COS—carbonyl sulphide) and nitrogen. Most of these substances are also targeted in biomethane or hydrogen matrices, but the thresholds are less stringent than those for hydrogen used in fuel cell vehicles. As mentioned above, these species have different physical and chemical properties, including a wide range of boiling points. Accordingly, the presence of these species in the CO_2_ stream can have negative effects of a physical nature (affecting the thermodynamic and transport properties of the stream), chemical nature (corrosion), microbiological and toxic and ecotoxic nature (effects on human and animal health and on the environment). These effects have been reviewed in ISO/TR27921:2020.

Furthermore, measurement challenges arise from the physical properties of CO_2_ itself. The critical point of pure CO_2_ is close to the ambient temperature. Therefore, phase changes and multiphase conditions can occur at the sampling points. There may be a partitioning of species between the different phases, and if only the gas phase is analysed, the composition may be underestimated. The composition of the CO_2_ stream needs to be monitored along the CCS chain downstream of the capture plant and the CO_2_ stream should be in a single phase at the time of sampling. The technical document only mentions sampling in Sulfinert cylinders, but also notes that it is important to address the standardisation of sampling and analysis procedures, as there is currently no standard for sampling matrices in which CO_2_ is the main component.

Monitoring and reporting guidelines (MRG) for CCS have been produced in 2010 as part of the CATO2 project [41], describing the monitoring and quantification of CO_2_ streams in CCS. Some guidance is given on sampling for CO_2_ concentration. For compressed CO_2_ flows, it is suggested to perform extractive sampling by pressure reduction and subsequent measurements of gaseous CO_2_ concentration at or near atmospheric pressure. The pressure reduction system in the sampling line should be designed to avoid liquid or droplet formation, e.g. by using a heated system. With such a sampling system, CO_2_ samples can then be collected in gas cylinders or sampling bags and even on sorption tubes. As for biomethane, the composition of the CO_2_ stream may not be known at the time of sampling, and similar problems may occur with flowmeters that are normally calibrated for a fixed composition.

### Hydrogen-enriched gas

Mixing hydrogen into existing natural gas pipelines is proposed as a means to increase the performance of renewable energy systems and as a means to deliver pure hydrogen to markets [42] (the hydrogen is extracted from the natural gas close to the point of use). Appropriate hydrogen concentrations are determined taking into account the risks associated with the utilisation of the gas in end-use appliances, public safety and the durability and integrity of the existing pipeline network.

At present [43], it is not possible to specify a limit value for hydrogen that would generally apply to all parts of the European gas infrastructure, as further studies need to be carried out to assess the impact of hydrogen in porous rock underground storage tanks, steel tanks in natural gas vehicles, gas turbines and gas engines [44]. The European Committee for Standardization (CEN) developed the standard EN 16,726:201 “Gas infrastructure—Quality of gas—Group H” [43]. The permissible hydrogen concentrations in natural gas systems are addressed separately in Annex E (informative). For sampling, EN16726 refers to ISO10715 [20] which is primarily intended for natural gas.

The ISO16726 standard specifies the relative density requirements (which, if determined according to ISO6976 [45], requires the determination of the composition of the hydrogen-enriched natural gas in terms of methane, hydrogen, other alkanes and nitrogen), for oxygen, for carbon dioxide and for sulphur species such as H_2_S, COS and mercaptans.

The sampling system (sampling line and sampling vessel) must be adapted to the geometry of the pipeline (e.g. its diameter) and the conditions (e.g. pressure). At high pressure, a probe is required, and cylinders are the only vessels mentioned, while at low pressure and small pipeline diameters, a probe may not be required and sampling in bags and on sorbent tubes is possible.

For all gases considered in this study, the sampling of high-pressure gases or gases containing reactive substances raises material issues. For the selection of materials for natural gas sampling, the standard ISO10715 [20] refers to a table from another standard, ISO16664 [46], which provides material compatibility for a list of gases such as inert gases, oxygen, carbon dioxide, carbon monoxide, alkanes, alkenes, aromatics, nitrogen oxides, chlorine, hydrogen chloride, ammonia, hydrogen sulphide and sulphur dioxide and for a list of materials (stainless steel, copper/brass, Hastelloy/monel/nickel, aluminium, polytetrafluorethene, polyether-ketone silica-lined, glass/quartz, fluorinated ethene-propene and silica-lined stainless steel).

According to this table, stainless steel is suitable for most of the impurities mentioned, except for CO at a mole fraction above 1% (of limited suitability) and for Cl_2_ and HCl at all (of limited suitability) and for H_2_S at a mole fraction below 0.001% (not suitable). Aluminium is also suitable for many of the impurities. However, there is no experience for NO_2_ with a mole fraction below 1% (aluminium is not suitable for NO_2_ with a mole fraction above 1%), for Cl_2_ with a mole fraction between 0.001 and 1% (aluminium is not suitable for Cl_2_ with a mole fraction above 1%) and for NH_3_. Aluminium is not suitable for HCl at mole fractions above 0.001%. Hastelloy/monel/nickel, glass/quartz and silica-lined stainless steel are suitable for all impurities in the three ranges of mole fractions (< 0.001%, > 0.001 to 1%, > 1%). The ISO10715 standard [20] even recommends analysing reactive compounds on site using direct sampling methods when practical, as even coated cylinders cannot eliminate the risk of absorption of reactive species.

## Conclusions

The purpose of this study is to identify the challenges and current limitations of sampling methods for the quality assessment of biomethane, hydrogen, carbon dioxide and hydrogen-enriched natural gas. The main challenge common to all these gases is to adequately manage the risk of impurities being lost by adsorption into the walls of the gas cylinders (or bags) and onto the sorbents either immediately or during transport to the analytical laboratory.

Materials in contact with gases that may contain reactive impurities should be impermeable to all species and should have a minimum of sorption and chemical inertness to the constituents being transferred. The same considerations apply to all parts of the sampling line and especially to those parts where pressure reduction takes place (regulators, valves, etc.). The material of the cylinder is probably even more critical when sampling hydrogen used as fuel in FCEVs, as the thresholds required in EN17124 and ISO14687 are very strict (low or sub-µmol/mol).

Performing “total” measurements (total hydrocarbons, total sulphur compounds, halogenated compounds for hydrogen, siloxanes and sulphur for biomethane, sulphur species for carbon dioxide and hydrogen-enriched natural gas) is another challenge to overcome in sampling, partly because of the wide range of boiling points of the compounds belonging to these families. For example, the existing sorbent tubes are not universal enough to trap all possible impurities of a given family.

Other challenges arise from the need for flow measurement specifically for enrichment methods and especially for biomethane, hydrogen-enriched natural gas and even carbon dioxide, for which the exact composition may not be known until a complete analysis is performed in a laboratory. Many flow meters are calibrated for a fixed composition and changes in composition drastically affect the flow measurement.

Finally, a more carbon dioxide–specific challenge arises from the physical properties of CO_2_ itself, as the critical point of pure CO_2_ is near ambient temperature, so that phase change and consequently multiphase conditions can occur at sampling points if the necessary precautions (such as heating the sampling line) are not taken.

As material compatibility issues are often mentioned but not well demonstrated experimentally, it is of great importance to increase the knowledge of adsorption effects of relevant species on different materials under different conditions (matrix, pressure, concentration). This could be done by more systematic recovery experiments (at the time of sampling) and short-term stabilities (over a period of time corresponding to the time it takes for the sample to be delivered to the analytical laboratory) at defined and relevant conditions in terms of pressure, matrix and concentration).

The results of these investigations should be compiled in material compatibility tables to assist industry in selecting suitable materials for cylinders and sampling lines.

## References

[1] The future of hydrogen, Technology report, The future of hydrogen – analysis - International Energy Agency, 2019.

[2] Gustafsson M, Svensson N (2021). Cleaner heavy transports – environmental and economic analysis of liquified natural gas and biomethane. J Clean Prod.

[3] Gardner D. Hydrogen production from renewable, Hydrogen production from renewables - Renewable Energy Focus [Online]. Available: www.renewableenergyfocus.com/view/3157/hydrogen-production-from-renewables. Accessed 15 11 2021.

[4] About biogas and biomethane, European Biogas Association (EBA), [Online]. Available: www.europeanbiogas.eu/about-biogas-and-biomethane. Accessed 16 11 2021.

[5] How much carbon dioxide is produced when different fuels are burned?. American Geosciences Institute; 2020. [Online]. Available: 25.

[6] Leung DY, Caramanna G, Maroto-Valer MM (2014). An overview of current status of carbon dioxide capture and storage technologies. Renew Sust Energy Rev.

[7] Almamoori A, Rownaghi AA, Krihnamurthy A, Rezaei F (2017). Carbon capture and utilization update. Energy Technol.

[8] Boot-Handford ME, Abanades C, Anthony EJ, Blunt MJ, Bradani S, Mac Dowel SN, Fernandez JR, Ferrari MC, Gross R, Hallett JP, Haszeldine RS, Heptonstall P, Lyngfelt A, Makuch AZ, Mangano E, Porter WT, Pourkashanian M, Rochelle GT, Shah N, Yao JG, Fennell PS (2015). Carbon capture and storage update. J Environ Sci.

[9] Carbon dioxide recovery and utilization. In: Aresta M, editor. Dordrecht: Kluwer Academic; 2003.

[10] Hu B, Guild C, Suib SL (2013). Thermal, electrochemical and photochemical convertion of CO2 to fuels and value-added products. J CO2 Util.

[11] Aresta M, Dibenedetto A, Angelini A (2014). Catalysis for the valorization of exhaust carbon: from CO2 to chemicals, materials and fuels. Technological use of CO2. Chem Rev..

[12] Chapter: 5 Biological utilization of CO2 into chemicals and fuels. In: Gaseous carbon waste streams utilization: status and research needs. Washington, DC: The National Academies of Sciences Engineering Medicine; 2019.

[13] EN17124:2019 Hydrogen fuel—product specification and quality assurance—proton exchange membrane (PEM) fuel cell applications for road vehicles. Bruxelles, Belgium: European Commite on Standardisation; 2019.

[14] ISO14687:2019 Hydrogen fuel quality – product specification. Geneva, Switzerland: ISO, International Organization for Standardization; 2019.

[15] EN16723–1:2016 Natural gas and biomethane for use in transport and biomethane for injection in the natural gas network, Part 1: Specifications for biomethane for injection in the natural gas network. Bruxelles, Belgium: European Commite on Standardisation; 2016.

[16] EN16723–2:2017 Natural gas and biomethane for use in transport and biomethane for injection in the natural gas network, Part 2: Automotive fuels specification. Bruxelles, Belgium: European Commite on Standardisation; 2017.

[17] ISO TR 27921:2020 Carbon dioxide capture, transportation, and geological storage – cross cutting issues – CO2 stream composition. Geneva, Switzerland: ISO, International Organization for Standardization; 2020.

[18] Arrhenius K, Brown AS, van der Veen AMH (2016). Suitability of different containers for the sampling and storage of biogas and biomethane for the determination of the trace-level impurities–a review. Anal Chim Acta.

[19] ISO21087:2019 Gas analysis – analytical methods for hydrogen fuel – proton exchange membrane (PEM) fuel cell applications for road vehicles. Geneva, Switzerland: ISO, International Organization for Standardization; 2019.

[20] ISO10715:1997 Natural gas – sampling guidelines. Geneva, Switzerland: ISO, International Organization for Standardization; 1997.

[21] Arrhenius K, Engelbrektsson J, Yaghooby H (2016). Development of analytical methods to gain insight into the role of terpenes in biogas plants. J Anal Bioanal Techn.

[22] Arrhenius K, Yaghooby H, Rosell L, Büker O, Culleton L, Bartlett S, Murugan A, Brewer P, Li J, van der Veen AMH, de Krom I, Lestremau F, Beranek J (2017). Suitability of vessels and adsorbents for the short-term storage of biogas/biomethane for the determination of impurities – siloxanes, sulfur compounds, halogenated hydrocarbons, BTEX. Biomass Bioenerg.

[23] Metrology for biogas, European Metrology Research Programme ENG54, 2014–2017, EURAMET, [Online]. Available: www.euramet.org/research-innovation. Accessed 05 07 2021.

[24] Gas sampling bags - cost-effective alternatives for air sampling, Restek, [Online]. Available: https://www.restek.com/globalassets/pdfs/literature/evss1335b-unv.pdf. Accessed 06 07 2021.

[25] Metrology for biomethane, European Metrology Programme for Innovation and Research, EURAMET, [Online]. Available: https://www.euramet.org/research-innovation/search-research-projects/details/project/metrology-for-biomethane/. Accessed 20 10 202.

[26] Woolfenden E (2010). Sorbent-based sampling methods for volatile and semi-volatile organic compounds in air. Part 2 - Sorbent selection and other aspects of optimising air monitoring methods. J Chromatogr A.

[27] Ramirez N, Marcé RM, Borrull F (2011). Determination of volatile organic compounds in industrial wastewater plant air emissions by multi-sorbent adsorption and thermal desorption-gas chromatography-mass spectrometry. Int J Environ Anal Chem.

[28] Using sorbent tubes – Choosing tubes and sorbents, [Online]. Available: www.markes.com. Accessed 05 07 2021.

[29] Arrhenius K, Büker O (2021). Comparison of different models to calculate the viscosity of biogas and biomethane in order to accurately measure flow rates for conformity assessment. Sci Rep.

[30] Arrhenius K, Fischer A, Büker O (2019). Methods for sampling biogas and biomethane on adsorbent tubes after collection in gas bags. Appl Sci.

[31] Deliverable 1.1.2 - Mixtures of siloxanes in matrices of methane and synthetic biogas validated using GC-FID, GC-AED and GC-MS and methods validated, Metrology for biogas, EURAMET EMRP ENG54, 2017.

[32] Arrhenius K, Aarhaug T, Bacquart T, Morris A, Bartlett S, Wagner L, Blondeel C, Gozlan B, Lescornez Y, Chramosta N, Spitta C, Basset E, Nouvelot Q, Rizand M (2021). Strategies for the sampling of hydrogen at refueling stations for purity assessment. Int J Hydrogen Energy.

[33] Barone G, Smith D, Higgins M. Characterizing the performance of surface modifications that enhance sensitivity, reliability, reproducibility and accuracy of analytical instruments, SilcoTek. [Online].

[34] A chromatographic view of inert coating of components used for sample transfer and holding, SilcoTek, [Online]. Available: https://www.silcotek.com/hubfs/LiteratureCatalog/E-books/%23EBK-UG-002AnIntroductiontoInertCoatings.pdf. Accessed 05 07 2021.

[35] Squire G. The performax cylinder passivation: a brand new approach for aluminium cylinders. In: Gas Analysis conference, Netherlands; 2017.

[36] Arrhenius K, Bartlett S. Deliverable 4.4.5 Good practice on the suitability of vessels and gas cylinders for sampling, hydrogen as required by ISO14867, EURAMET Metrology for hydrogen vehicles EMPIR 16ENG01. 2020.

[37] ISO14687:2019 Hydrogen fuel quality – product specification. Geneva, Switzerland: International Organization for Standardization (ISO); 2019.

[38] Arrhenius K, Büker O, de Krom I, Heikens D, van Wijk J (2020). Hydrogen purity analysis: suitability of sorbent tubes for trapping hydrocarbons, halogenated hydrocarbons and sulphur compounds. App Sci.

[39] Metrology for hydrogen vehicles 2, EURAMET EMPIR 19ENG04, 2020–2023.

[40] ISO TR 27921:2020 Carbon dioxide capture, transportation, and geological storage – cross cutting issues – CO2 stream composition. Geneva, Switzerland: International Organization for Standardization (ISO); 2020.

[41] The CATO-2 programme, [Online]. Available: https://www.co2-cato.org/cato. Accessed 05 07 2021.

[42] The effect of hydrogen injection in natural gas networks for the Dutch underground storages. Netherlands Entreprise Agency.

[43] EN 16726:2015 Gas infrastructure – quality of gas – group H, European Commite on Standardisation. Bruxelles, Belgium: European Committee on Standardization; 2015.

[44] Altfeld K, Pinchbeck D. Admissible hydrogen concentrations in natural gas systems. The European Gas Research Group (GERG); 2013.

[45] ISO6976:2016 Natural gas – calculation of calorific values, density, relative density and Wobbe indices from composition. Geneva, Switzerland: International Organization for Standardization (ISO); 2016.

[46] ISO16664:2017 Gas analysis – handling of calibration gases and gas mixtures – guidelines. Geneva, Switzerland: International Organization for Standardization (ISO); 2017.

